# Neuroprotective Effects of Betanin in a Mouse Model of Parkinson’s Disease: Behavioural and Neurotransmitter Pathway Insights

**DOI:** 10.3390/ijms26199726

**Published:** 2025-10-06

**Authors:** Katarzyna Ziętal, Kamilla Blecharz-Klin, Ilona Joniec-Maciejak, Agnieszka Piechal, Justyna Pyrzanowska, Ewa Machaj, Dagmara Mirowska-Guzel, Ewa Widy-Tyszkiewicz

**Affiliations:** Department of Experimental and Clinical Pharmacology, Centre for Preclinical Research and Technology CePT, Medical University of Warsaw, Banacha 1B, 02-097 Warsaw, Poland; katarzyna.zietal@wum.edu.pl (K.Z.); ilona.joniec-maciejak@wum.edu.pl (I.J.-M.); agnieszka.piechal@wum.edu.pl (A.P.); justyna.pyrzanowska@wum.edu.pl (J.P.); ewa.machaj@wum.edu.pl (E.M.); dagmara.mirowska-guzel@wum.edu.pl (D.M.-G.); etyszk@gmail.com (E.W.-T.)

**Keywords:** betanin, behaviour, CNS, neurotransmission, Parkinson’s disease model

## Abstract

The study aimed to evaluate the effect of betanin—a bioactive, natural pigment found in beetroot and prickly pear—on cognitive function, motor performance, and neurotransmission in a mouse model of Parkinson’s disease (PD). Aged mice with PD-like symptoms induced by 1-methyl-4-phenyl-1,2,3,6-tetrahydropyridine (MPTP) were pretreated with betanin (50 or 100 mg/kg b.w./day) via drinking water. Behavioural tests assessed motor skills, anxiety-related behaviour, and spatial memory. Biochemical analyses of central nervous system structures were conducted using high-performance liquid chromatography (HPLC) to determine neurotransmitter levels and metabolites. Betanin improved motor and cognitive functions in MPTP-treated mice. While learning ability remained unchanged, the 50 mg/kg dose alleviated spatial memory deficits. Biochemically, betanin moderately limited dopamine depletion and significantly influenced dopamine metabolism and serotonin levels. These findings suggest that betanin, as a functional food component, may exert neuroprotective effects and support cognitive and motor function in neurodegenerative conditions such as PD.

## 1. Introduction

Betanin is a natural component found in beetroot and prickly pear fruit, known for its potential antioxidant, antibacterial, antiviral, and anticancer properties. The broad biological activity of betanin occurs at many levels and has great potential for health benefits [1,2,3,4,5,6,7]. The neuroprotective effect of betanin was confirmed by in vitro studies conducted by Hadipour et al. [8]. It was shown that pretreatment with betanin (20 and 50 µM) attenuated oxidative stress induced by 6-hydroxydopamine and H_2_O_2_ in PC12 cells (a model of Parkinson’s cell damage). Betanin improved cell viability by increasing the number of surviving cells and phosphoinositide 3-kinase (PI3K) phosphorylation, as well as reducing mitochondrial cytochrome complex release and Stress-Activated Protein Kinase/c-Jun N-terminal Kinase (SAPK/JNK) phosphorylation. These changes then contribute to the inhibition of caspase 9 and 3 activity, which may ultimately prevent or delay the progression of neural death observed in Parkinson’s disease. Ahmadi et al. [9] demonstrated the anti-inflammatory effect of betanin by significantly inhibiting tumour necrosis factor-alpha (TNF-α), interleukin-6 (IL-6), and interleukin-1 beta (IL-1β). Furthermore, gene regulatory activity (e.g., effect on nuclear factor erythroid 2–related factor 2 (Nrf2) -dependent signalling pathway), effect on DNA-damage prevention, sirtuin 1 cell signalling pathways, low-density lipoprotein antioxidant protection, induction of antioxidant and anti-inflammatory activities such as cyclooxygenase-2 inhibition of betanin were described [6,10,11,12,13].

Beetroot betalain pigments such as betanin and isobetanin have also been shown to reduce amyloid β (Aβ) aggregation and toxicity in a transgenic *Caenorhabditis elegans* model that expresses human Aβ42 protein [14].

The neuroprotective effect of betanin was also confirmed in animals with injury caused by ischemia and reperfusion. Thong-Asa et al. [15] two-week pretreatment with betanin (100 mg/kg b.w. p.o.) resulted in significant reduction in cerebral infarction, malondialdehyde (MDA) level and white matter pathology in cerebral ischemia–reperfusion injury as found in stroke. Tural et al. [16] demonstrated in a spinal cord ischemia–reperfusion model that betanin treatment significantly reduced MDA levels and myeloperoxidase (MPO) activity, while increasing glutathione (GSH) levels. The neuroprotective effect of betanin also included inhibition of lipid peroxidation, as well as increasing catalase activity and enhancing the effect on reduced glutathione [17]. All these processes play a key role in preventing and limiting neurodegenerative changes.

The assessment of the potential neuroprotective effect of betanin is important due to the lack of effective pharmacotherapy of such neurodegenerative diseases such as Parkinson’s disease, Alzheimer’s disease, and post-stroke conditions. Therefore, the possibility of influencing them with betanin is a very promising direction for further research.

The data on betanin published to date are pioneering and require continuation and expansion of knowledge based on in vivo tests. The aim of this study was to assess the effects of betanin on cognition, motor functions, anxiety responses, motor coordination, and neurotransmission in a mouse model of Parkinson’s disease induced by MPTP.

The evaluation of the effect of betanin on the improvement of motor and cognitive deficits in an experimental model of neurodegenerative disease confirms its promising role in neuroprotection.

## 2. Results

### 2.1. Impact on Body Weight

**Figure 1:**
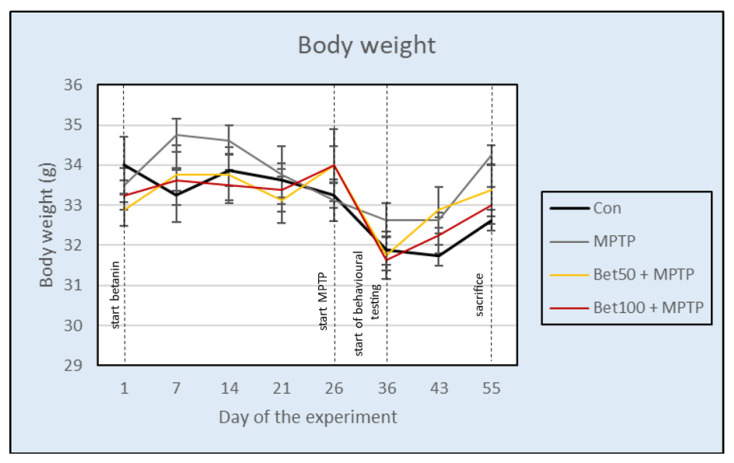
Effect of betanin and MPTP administration on the body weight of animals (g) during the experiment.

During the experiment, no significant differences in body weight were observed between the control and treatment groups (F(3,28) = 0.89, *p* > 0.05). The body weight of animals was temporarily reduced when intraperitoneal administration of MPTP (groups: MPTP, Bet50 + MPTP and Bet100 + MPTP) or NaCl solution (Con) was started, but then the body weight of animals increased and stabilized (Figure 1). These results suggest that neither MPTP or betanin treatment caused lasting changes in body weight of the experimental animals.

### 2.2. Influence on Motor Coordination and Balance

**Figure 2:**
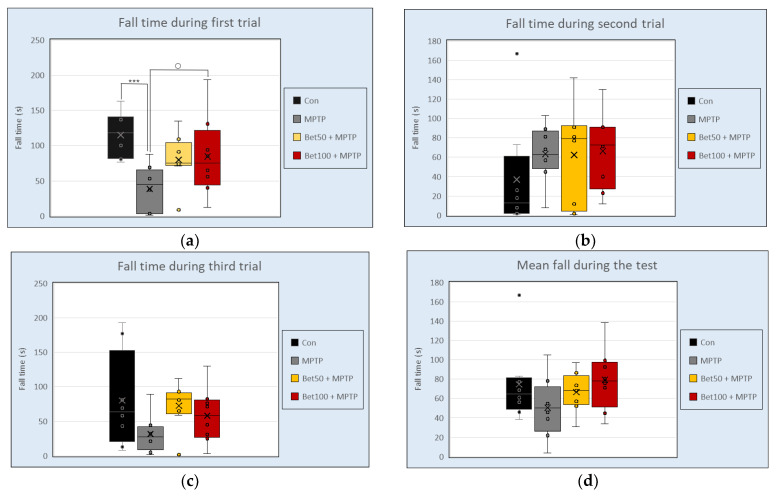
Falling time (s) during three attempts (**a**–**c**) and mean falling time (s). (**d**) in Rota Rod test for control group (Con, n = 8), animals treated with MPTP alone (MPTP, n = 8), and betanin with MPTP (Bet50 + MPTP, n = 8; Bet100 + MPTP, n = 8). *** vs. Con, *p* < 0.005 (NK); ^○^ MPTP vs. Bet100 + MPTP, *p* < 0.05 (NIR). Horizontal line—median, x—mean.

The Rota Rod apparatus assessed the motor skills of mice. Experimental data analyzed with ANOVA showed significant differences between groups in the first trial (F(3,28) = 4.68, *p* < 0.05) (Figure 2a). Mice injected with MPTP had a shorter falling time (measured in seconds) on the rotating cylinder than the control group (*p* < 0.005, NK) and betanin at a dose of 100 mg (*p* < 0.05, NIR). There were no significant differences in falling time in the second trial (F(3,28) = 0.71) (Figure 2b), third trial (F(3,28) = 1.79) (Figure 2c), and mean falling time (F(3,28) = 1.70) (*p* > 0.05) (Figure 2d). These findings indicate that MPTP administration impaired motor coordination in mice, as evidenced by reduced performance in the first trial, while betanin at a dose of 100 mg alleviated this impairment, suggesting a protective effect. Performance in the first trial reflects baseline motor ability, while subsequent trials may indicate motor learning, fatigue, or adaptation to the increasing task difficulty.

### 2.3. Changes in Gait and Locomotor Patterns

**Figure 3:**
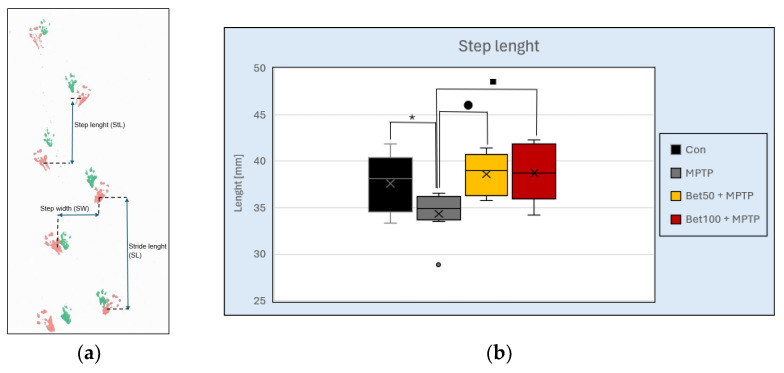
Representative footprint pattern used for gait analysis (**a**). Measured parameters are indicated: step length (distance between successive placements of opposite limbs), stride length (distance between successive placements of the same limb), and step width (distance between left and right paw placements). MPTP-treated mice showed a shortened step length pattern compared to the control and betanin groups (**b**). * vs. Con, *p* < 0.05 (NK); ^■^ MPTP vs. Bet50 + MPTP, *p* < 0.05 (NK); ^●^ MPTP vs. Bet100 + MPTP, *p* < 0.05 (NK). Horizontal line—median, x—mean.

Footprint analysis was performed to assess gait alterations induced by MPTP and the potential protective effects of betanin. As shown in Figure 3a, key gait parameters—step length, stride length, and step width—were measured to evaluate motor coordination and locomotor deficits. There were statistically significant differences in step length between the mouse groups (F(3,28) = 4.49, *p* < 0.05). In the MPTP group, mice showed an abnormal shortened step length pattern compared to the control group (*p* < 0.05, NK) (Figure 3b). Betanin reversed the shortening effect induced by MPTP (*p* < 0.05, NK). No statistically significant differences were observed in stride length (F(3,28) = 0.863, *p* > 0.05) or step width (F(3,28) = 0.558, *p* > 0.05) between the control group and mice from the other experimental groups. These results demonstrate that MPTP disrupts normal gait by shortening step length, while betanin treatment effectively mitigates this impairment, restoring gait parameters to control levels.

### 2.4. Behavioural Outcomes Related to Anxiety

**Figure 4:**
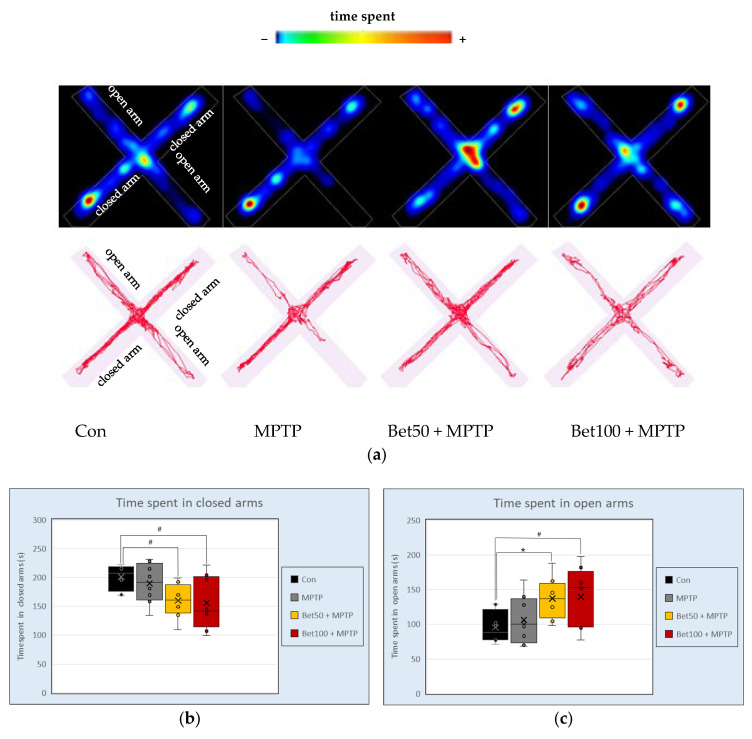
Representative movement trajectories during the Elevated Plus Maze (EPM) test: heatmaps and trace plots (**a**) showing the activity of control (Con, n = 8), MPTP-treated (MPTP, n = 8), and MPTP plus betanin-treated animals (Bet50 + MPTP, n = 8; Bet100 + MPTP, n = 8). Time (s) spent in the closed (**b**) and open (**c**) arms of the apparatus. * vs. Con, *p* < 0.05 (NK); # vs. Con, *p* < 0.05 (NIR). Horizontal line—median, x—mean.

In the elevated Plus Maze (EPM), analysis of variance revealed differences between groups in time spent in closed (F(3,28) = 3.37), and open arms (F(3,28) = 3.46; *p* < 0.05). Mice given MPTP in combination with betanin at both doses spent less time in the closed arms of the apparatus than animals from the control group (*p* < 0.05, NIR) (Figure 4b). Opposite effects were observed in time spent in the open arms of the apparatus—in the control group compared to betanin, both doses 50 mg (*p* < 0.05, NK) and 100 mg (*p* < 0.05, NIR) (Figure 4c). Representative tracking data and heat maps corroborated the quantitative results, indicating more pronounced exploration of the open arms of the EPM apparatus by the betanin-treated groups (Figure 4a). Concurrently, preliminary visual assessment of the graphical data suggested considerable variability within certain groups, particularly concerning the time spent in the open arms. However, this observed variability was not statistically confirmed. Although visual representations—such as movement trajectories and heat maps—indicated a trend toward prolonged time spent in the open arms by animals in the betanin-treated groups, including compared to the MPTP group, which was also reflected in mean values, these differences did not reach statistical significance and were therefore classified as a non-confirmed tendency. These observations underscore the complexity of behavioural outcomes in neurodegenerative models and highlight the importance of considering intra-group variability alongside mean differences when interpreting the data.

During EPM there were no statistically significant differences between groups in the number of entries into open arms (F(3,28) = 1.05) and closed arms (F(3,28) = 1.27), total distance (F(3,28) = 2.11), average speed (F(3,28) = 2.05), as well as in the waiting time to the first entry into open arms (F(3,28) = 0.93) (*p* > 0.05). These findings suggest that betanin administration at both tested doses enhances anxiolytic behaviour without affecting general locomotor activity.

### 2.5. Alterations in Exploratory Activity and Locomotion

**Figure 5:**
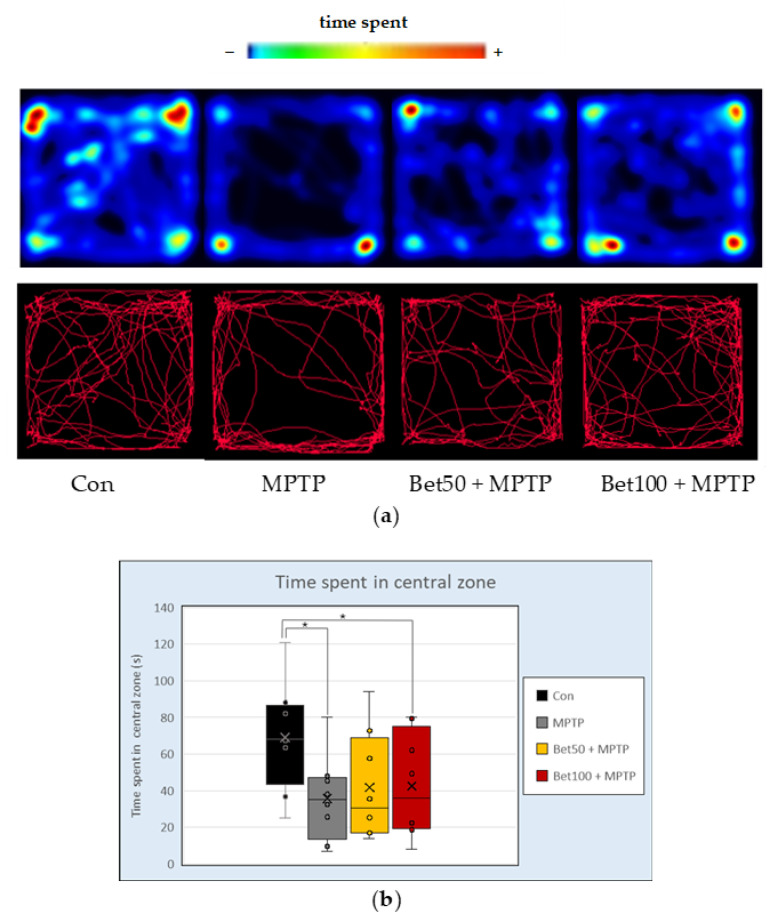
Representative heatmaps and movement trajectories (**a**) from the Open Field (OF) test in mice from the control (Con, n = 8), MPTP-treated (MPTP, n = 8), and betanin plus MPTP-treated groups (Bet50 + MPTP, n = 8; Bet100 + MPTP, n = 8). (**b**) Time (s) spent in the central zone of the apparatus during the OF test in the same groups. * vs. Con, *p* < 0.05 (NK). Horizontal line—median, x—mean.

The Open Field test allows for the assessment of anxiety and locomotion of mice. No statistically significant differences were found between groups in the following parameters: climbing behaviour (F(3,28) = 1.16), time spent moving (F(3,28) = 0.91), total distance travelled (F(3,28) = 0.64), mean speed (F(3,28) = 0.64), latency to first entry into the central zone (F(3,28) = 0.88), time spent immobile (F(3,28) = 0.38), and defecation (F(3,28) = 0.38) (all *p* > 0.05). In contrast, statistical analysis (ANOVA; F(3,28) = 3.20, *p* < 0.05) revealed a significant difference in the time spent in the central zone of the arena. Control animals spent significantly more time in the centre compared to MPTP-treated mice and those receiving MPTP in combination with a higher dose of betanin (100 mg) (*p* < 0.05, NK; Figure 5b). These results were visually supported by representative tracking plots and heat maps, which showed markedly reduced central zone exploration, particularly in the MPTP-treated group (Figure 5a). Although no statistically significant improvement was observed in the groups co-treated with betanin, visual inspection of the tracking data suggests a trend toward increased activity in the central zone in animals receiving betanin. These observations may point to a potential effect of betanin; however, as they were not statistically confirmed in the present analysis, they should be interpreted with caution.

### 2.6. Effects on Spatial Learning and Memory

**Figure 6:**
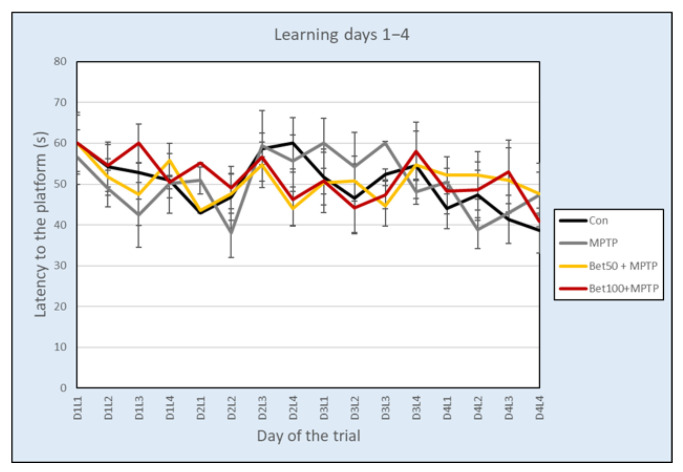
Latency (mean ± SEM) associated with learning the platform location in the Water Maze test for mice from the control (Con, n = 8), MPTP-treated (MPTP, n = 8) and MPTP + betanin (Bet50 + MPTP, n = 8; Bet100 + MPTP, n = 8) groups.

During the acquisition phase of Water Maze (days 1–4, trials 1–16) there were no statistically significant differences in the latency to learn the platform location between the control group and the betanin and MPTP-treated mice, as assessed by repeated measures analysis of variance (F(3,28) = 0.808, *p* > 0.05) (Figure 6). Experimental data analyzed with ANOVA revealed no differences in the mean speed (F(3,28) = 0.257, *p* > 0.05) and path (F(3,28) = 0.547, *p* > 0.05) during the acquisition phase.

**Figure 7:**
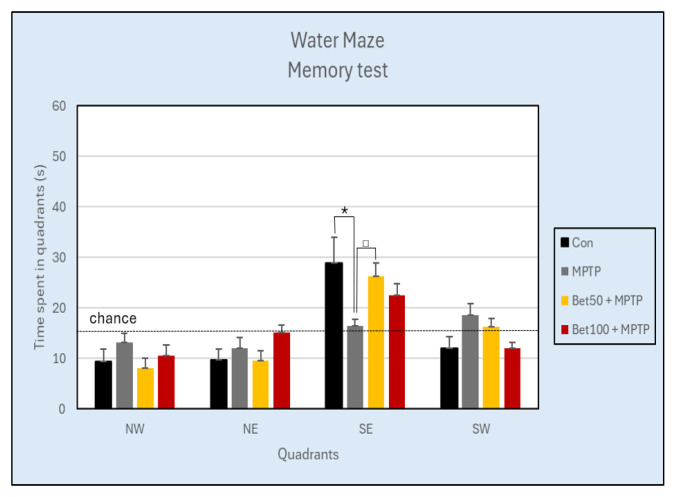
Time (s) spent in each of the four quadrants during the Water Maze Memory test (day 5) of the control group (Con, n = 8), and animals with the neurotoxin MPTP alone, (MPTP, n = 8), and with the combination of betanin (Bet50 + MPTP, n = 8; Bet100 + MPTP, n = 8). * vs. Con, *p* < 0.05 (NK), ^□^ MPTP vs. Bet50 + MPTP, *p* < 0.05 (NIR). The horizontal line represents the chance level (25%), which reflects the expected performance if the animal were swimming randomly without spatial memory of the platform location.

Analysis of the results using ANOVA revealed no significant differences in the number of crossings over the previous platform position in the SE quadrant during the memory test (day 5, trial 17) (Con: 2.63 ± 0.42; MPTP: 0.75 ± 0.31; Bet50 + MPTP: 2.38 ± 0.75; Bet100 + MPTP: 1.63 ± 0.56) (F(3,28) = 2.45, *p* > 0.05), however statistically significant differences were confirmed in the time spent in the SE quadrant during the memory test (F(3,28) = 2.99, *p* < 0.05). MPTP-intoxicated mice spent less time in the SE zone compared to the control group (*p* < 0.05, NK) and the group with a lower dose of betanin (*p* < 0.05, NIR). In the remaining three quadrants of the pool: NW (F(3,28) = 1.05), NE (F(3,28) = 1.80) and SW (F(3,28) = 2.86) no significant differences were found between the experimental groups (*p* > 0.05) (Figure 7).

There were no differences in either the distance travelled (F(3,28) = 1.04) or the speed (F(3,28) = 1.03) (*p* > 0.05).

In the visible test, there were no statistically significant differences in latency to the previous platform location (F(3,28) = 0.74), mean speed (F(3,28) = 0.79) or path (F(3,28) = 1.10) (*p* > 0.05).

The Water Maze results suggest that spatial learning was not significantly affected by MPTP or betanin treatment during the acquisition phase, while MPTP intoxication reduced memory retention in the memory test, with betanin treatment showing a protective trend.

### 2.7. Significant Changes in Monoamine Concentration in Selected Brain Regions

Monoamine concentration in selected CNS structures and their turnover are presented in Tables S1–S4 (Supplementary Materials).

#### 2.7.1. Dopamine (DA) and Metabolites

**Figure 8:**
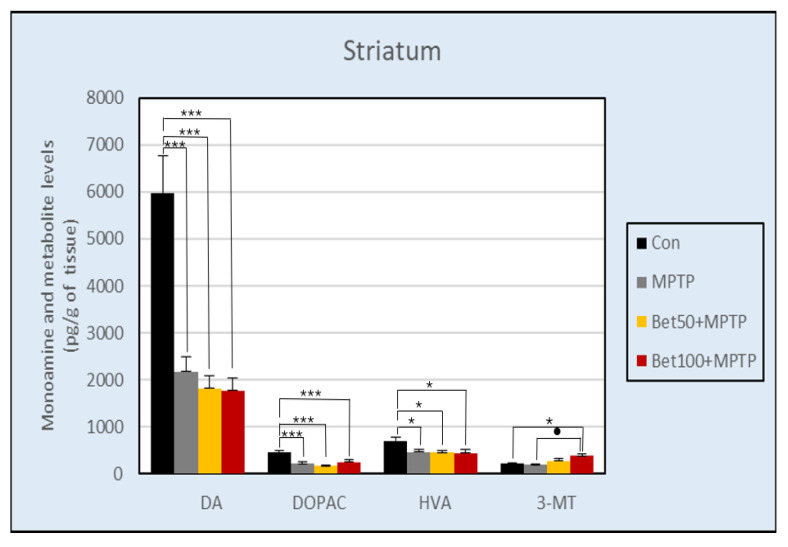
Concentration of DA and its metabolites: DOPAC, HVA and 3-MT in the striatum of mice from the control group, MPTP injected group and animals treated with MPTP in combination with betanin. Data represent means ± SEM of 8 animals in each group. * vs. Con, *p* < 0.05 (NK); *** vs. Con, *p* < 0.005 (NK); ● MPTP vs. Bet100 + MPTP, *p* < 0.05 (NK).

The results of the ANOVA revealed a statistically significant effect of treatment on DA content in the striatum (F(3,28) = 18.87), hippocampus (F(3,28) = 4.61) and hypothalamus (F(3,28) = 3.29) (*p* < 0.05). In the striatum, dopamine levels were higher in the control group compared to all animals in the Parkinson’s disease model: MPTP alone and in combination with betanin (*p* < 0.005, NK) (Figure 8). The same results were in the hypothalamus (Supplementary Material—Table S2): control group had higher levels of dopamine compared to groups with: MPTP, 50 mg of betanin (*p* < 0.05, NK) and 100 mg of betanin (*p* < 0.05, NIR). In the hippocampus, control group had higher levels of dopamine compared to MPTP (*p* < 0.05, NK), betanin at lower dose (*p* < 0.01, NK) and higher dose (*p* < 0.05, NK) hypothalamus (Supplementary Material—Table S1).

Statistical analysis did not show significant differences between groups in DA content in the prefrontal cortex (F(3,28) = 2.25) (Supplementary Material—Table S1), as well as cerebellum (F(3,28) = 1.95), spinal cord (F(3,28) = 2.28) and medulla oblongata (F(3,28) = 1.03) (*p* > 0.05) (Supplementary Material—Table S2).

Statistically significant differences were also demonstrated between the content of dopamine metabolite 3,4-dihydroxyphenylacetic acid (DOPAC) in the striatum (F(3,28) = 8.13) (Figure 8), and hypothalamus (F(3,28) = 3.81)(*p* < 0.05) (Supplementary Material—Table S2). In the striatum, the control group had a significantly higher level of DOPAC compared to all groups intoxicated with MPTP (*p* < 0.005, NK). In the hypothalamus, the level of DOPAC was also significantly higher in the control group compared to all other groups (*p* < 0.05, NK). The lack of statistical differences between groups was confirmed in DOPAC content in the hippocampus (F(3,28) = 2.81) and prefrontal cortex (F(3,28) = 0.60) (Supplementary Material—Table S1), as well as and medulla oblongata (F(3,28) = 1.12) (*p* > 0.05) (Supplementary Material—Table S2).

At the same time, differences were found in the level of another DA metabolite—HVA (homovanillic acid) in the prefrontal cortex (F(3,28) = 3.08) (Supplementary Material—Table S1), striatum (F(3,28) = 3.94) (Figure 8), and hypothalamus (F(3,28) = 4.99) (*p* < 0.05) (Supplementary Material—Table S2). The level of HVA in the prefrontal cortex was significantly higher in the group receiving betanin at a dose of 50 mg (*p* < 0.05, NIR), and 100 mg (*p* < 0.05, NK) compared to the MPTP group. In the striatum, the HVA level was significantly higher in the control group compared to all other groups (*p* < 0.05, NK) (Figure 8). In the hypothalamus, the same results were obtained as in the striatum: the control group had a higher ratio than betanin (*p* < 0.05, NK) and MPTP (*p* < 0.01, NK) group (Supplementary Material—Table S2). ANOVA analysis showed no statistically significant differences between groups in HVA content in the hippocampus (F(3,28) = 1.62, *p* > 0.05) (Supplementary Material—Table S1).

Differences in 3-MT (3-metoxytyramine) content were detected in the striatum (F(3,28) = 3.88) (Figure 8) and hippocampus (F(3,28) = 3.74) (*p* < 0.05) (Supplementary Material—Table S1). The level of this dopamine metabolite in the striatum was higher in mice treated with betanin at a dose of 100 mg compared to MPTP and the control group (*p* < 0.05, NK). In the hippocampus, the control group showed a higher content of 3-MT compared to the groups receiving betanin (*p* < 0.05, NK). There was no statistically significant difference in the level of 3-MT in the hypothalamus (F(3,28) = 2.79, *p* > 0.05) (Supplementary Material—Table S2).

Experimental data analyzed with ANOVA showed differences in DOPAC/DA turnover in prefrontal cortex (F(3,28) = 5.59) and striatum (F(3,28) = 3.97) (*p* < 0.05) (Supplementary Material—Table S3). In prefrontal cortex, a decrease in DA turnover (DOPAC/DA) was observed in animals treated with betanin: Bet50 + MPTP (*p* < 0.005, NK) and Bet100 + MPTP (*p* < 0.05, NK) compared to the control group. In striatum, higher dose of betanin had higher turnover compared to the control group, and animals received betanin in a lower dose (*p* < 0.05, NK). There was no statistically significant difference between groups in the DOPAC/DA ratio in the hippocampus (F(3,28) = 0.92) (Supplementary Material—Table S3), hypothalamus (F(3,28) = 0.78), and medulla oblongata (F(3,28) = 0.88) (*p* > 0.05) (Supplementary Material—Table S4).

The analysis showed that HVA/DA turnover (F(3,28) = 4.14, *p* < 0.05) was significantly lower in the control group compared with Bet50 + MPTP group (*p* < 0,01, NK) and Bet100 + MPTP group (*p* < 0.05, NIR) in the striatum (Supplementary Material—Table S3). HVA/DA turnover in the prefrontal cortex (F(3,28) = 2.11), hippocampus (F(3,28) = 1.95) and hypothalamus (F(3,28) = 1.99) did not change between groups (*p* > 0.05) (Supplementary Material—Tables S3 and S4).

#### 2.7.2. 5-Hydroxytryptamine (5-HT) and Metabolites

The results of the ANOVA revealed a statistically significant differences in serotonin levels in striatum (F(3,28) = 3.90), hippocampus (F(3,28) = 7.23), prefrontal cortex (F(3,28) = 3.36) (Supplementary Material—Table S1), hypothalamus (F(3,28) = 3.07), and spinal cord (F(3,28) = 3.65) (*p* < 0.05) of experimental mice (Supplementary Material—Table S2). 5-HT level in striatum was higher in animals treated with betanin at a dose of 50 mg (Bet50 + MPTP) compared to the MPTP group (*p* < 0.05, NK) and the control group (*p* < 0.05, NIR). It was also lower in the MPTP group compared to animals receiving a higher dose of betanin (Bet100 + MPTP)(*p* < 0.05, NIR). In the hippocampus, the Bet50 + MPTP group had higher 5-HT levels compared to the other experimental groups: the control group (*p* < 0.005, NK), group receiving only MPTP injection (*p* < 0.05, NK) and betanin at a dose of 100 mg (*p* < 0.01, NK).

In the prefrontal cortex, the analysis showed a significant decrease in the 5-HT content with the higher dose of betanin compared to the group receiving 50 mg of betanin (*p* < 0.05, NK) and the control group (*p* < 0.05, NIR). In the spinal cord, 5-HT levels were lower in the Bet100 + MPTP group compared to the Bet50 + MPTP and MPTP groups (*p* < 0.05, NK). In the hypothalamus, 5-HT levels were lower in animals given the higher dose of betanin compared to the control group (*p* < 0.05, NK) and Bet50 + MPTP (*p* < 0.05, NIR). Statistical analysis of the results did not show any significant differences between groups in 5-HT content in the cerebellum (F(3,28) = 1.02) and medulla oblongata (F(3,28) = 1.32) (*p* > 0.05) (Supplementary Material—Table S2).

Statistically significant differences were also shown in the level of the 5-HT metabolite—5-HIAA (5-hydroxyindoloacetic acid) in the prefrontal cortex (F(3,28) = 5.72) (Supplementary Material—Table S1), and spinal cord (F(3,28) = 3.18) (*p* < 0.05) (Supplementary Material—Table S2). Analysis of the prefrontal cortex showed an increased level of 5-HIAA in the Bet100 + MPTP group compared to other MPTP intoxicated groups (*p* < 0.05, NK) and the control group (*p* < 0.005, NK). In the spinal cord group, mice receiving betanin at a dose of 100 mg had a higher level of 5-HIAA compared to the control group (*p* < 0.05, NK).

Experimental data analyzed by ANOVA did not reveal any statistically significant differences between groups in the 5-HIAA ratio in the striatum (F(3,28) = 0.41), hippocampus (F(3,28) = 2.12), medulla oblongata (F(3,28) = 0.53), hypothalamus (F(3,28) = 0.87) and cerebellum (F(3,28) = 2.69) (*p* > 0.05) (Supplementary Material—Tables S1 and S2).

HPLC analysis showed a significant difference between serotonin turnover (5-HIAA/5-HT) in the prefrontal cortex (F(3,28) = 7.63) (Supplementary Material—Table S3), medulla oblongata (F(3,28) = 3.24), spinal cord (F(3,28) = 13.70), and hypothalamus (F(3,28) = 4.03) (*p* < 0.05) (Supplementary Material—Table S4). In the spinal cord and prefrontal cortex, a higher 5-HIAA/5-HT turnover was observed in the Bet100 + MPTP group compared to the other groups (*p* < 0.005, NK). In the medulla oblongata, the Bet100 + MPTP group had significantly higher serotonin turnover compared to the control group (*p* < 0.05, NK). The hypothalamus also showed such higher turnover in the Bet100 + MPTP group compared to the control group (*p* < 0.05, NK) and the Bet50 + MPTP group (*p* < 0.05, NIR).

No statistically significant differences in 5-HIAA/5-HT turnover between groups in the striatum (F(3,28) = 1.63), hippocampus (F(3,28) = 2.19) and cerebellum (F(3,28) = 0.15) (*p* > 0.05) were observed (Supplementary Material—Tables S3 and S4).

#### 2.7.3. Noradrenaline (NA) and Metabolites

Statistical analysis (ANOVA) indicated significant differences between groups in noradrenaline levels in the prefrontal cortex (F(3,28) = 4.84, *p* < 0.05) (Supplementary Material—Table S1). The control group had significantly higher NA levels compared to the betanin treated groups (Bet50 + MPTP: *p* < 0.05, NK; Bet100 + MPTP: *p* < 0.01, NK). The NA content was the same in the experimental groups in the hippocampus (F(3,28) = 0.07), striatum (F(3,28) = 1.34), cerebellum (F(3,28) = 0.67), spinal cord (F(3,28) = 0.61), medulla oblongata (F(3,28) = 0.33) and hypothalamus (F(3,28) = 0.95) (*p* > 0.05) (Supplementary Material—Tables S1 and S2).

The level of noradrenaline metabolite—MHPG (3-metoxy-4-hydroxyphenylglycol) in the medulla oblongata remained unchanged in all groups of mice (F(3,28) = 0.65) (*p* > 0.05, NK) (Supplementary Material—Table S2). MHPG was not detected in the remaining structures (Supplementary Material—Tables S1 and S2).

Statistical analysis of the results did not reveal significant differences in MHPG/NA turnover (F(3,28) = 0.648, *p* >0.05) in the medulla oblongata (Supplementary Material—Table S4).

## 3. Discussion

It is believed that bioactive components found in plants, such as betanin, can help maintain health and prevent degenerative diseases, especially in the early stages. According to current views, regular consumption of foods rich in betacyanins can lead to a reduction in the occurrence of the neurodegenerative diseases resulting from structural damage to CNS cells [18].

In this study, a mouse model of PD induced by MPTP was used to analyze the effect impact of betanin on behavioural parameters and neurotransmission in CNS structures. MPTP administration impaired motor coordination and gait in mice, as evidenced by reduced performance in initial motor trials and shortened step length, while betanin treatment effectively mitigated these deficits. Neurotoxin also induced anxiety-like behaviour and reduced exploratory activity without affecting general locomotion, effects that were alleviated by betanin in a dose-dependent manner.

MPTP is primarily known for inducing dopaminergic neurodegeneration and usually exacerbates anxiety-related behaviours [19]. However, its effects on anxiety can be complex and context-dependent. Some studies report that MPTP produces no obvious behavioural deficits under certain conditions [20] or may even increase exploratory behaviour in specific paradigms [19]. Betanin’s antioxidative and neuromodulatory properties, particularly its interaction with the serotonergic system, might reduce oxidative stress and inflammation within limbic regions, leading to a dose-dependent anxiolytic effect. This could explain why higher doses of betanin increase time spent in open arms despite MPTP administration. Further studies are needed to clarify the mechanisms underlying this modulation.

In this experiment spatial learning during the acquisition phase of the Water Maze was not significantly affected by either MPTP or betanin; however, MPTP impaired memory retention in the memory test, with betanin showing a protective trend.

In the behavioural tests, such as the EPM, visual inspection of the tracking data indicated considerable variability in the analyzed parameters within certain experimental groups. Nevertheless, despite apparent trends, the observed patterns did not reach statistical significance. This dispersion of results may be attributed to individual differences in motor coordination, muscle strength, and baseline activity levels among animals. Moreover, such variability is frequently reported in rodent models, especially in studies involving neurotoxins like MPTP. The response to MPTP can differ markedly between animals, even within the same treatment group, due to differences in metabolism, blood–brain barrier permeability, or inherent susceptibility of dopaminergic neurons. Additionally, behavioural tests such as the rotarod are highly sensitive to subtle individual traits, including levels of anxiety, motivation, and prior motor experience, which can further contribute to outcome variability. These factors highlight the importance of using appropriate statistical approaches and sufficient sample sizes when interpreting behavioural data in neurodegeneration models.

It is well known that intraperitoneal injection of MPTP leads to the formation of the toxic metabolite MPP+ with the participation of monoamine oxidase B (MAO-B), and in addition to striatal dopamine depletion and neurodegeneration, which produces symptoms mimicking PD [21]. In nonhuman primate models, MPTP has been shown to induce damage to both the caudate nucleus and the anterior cingulate cortex [22]. In Parkinson’s disease, neuronal damage is not limited to the striatum but also involves other tissues and neurotransmitter systems other than dopaminergic. In humans, early in the disease, there is a reduction in cortical serotonergic and catecholaminergic innervation of the frontal cortex. Masilamoni et al. [23] showed that chronic MPTP treatment causes longitudinal reductions in cortical serotonergic and catecholamine innervation in both motor-symptomatic and asymptomatic rhesus macaques (Macaca mulatta), but the striatum in asymptomatic animals treated with MPTP did not show any significant loss of serotonergic innervation. MPTP neurotoxicity is associated with severe oxidative stress, mitochondrial apoptosis, inflammatory excitotoxicity, and intraneuronal protein inclusion bodies formation.

In our study, the dopaminergic system clearly suffered from the application of the neurotoxin, because injection of MPTP caused a selective deprivation of dopaminergic neurons in the substantia nigra. This was manifested by a simultaneous decrease in the level of dopamine and its metabolites not only in the striatum but also in the hippocampus and hypothalamus. These damages were so severe that the administration of betanin only tended to reduce (without statistical significance) the toxic effects on the dopaminergic system. Despite such profound changes, betanin administration at a dose of 100 mg was able to increase the concentration of 3-MT in the striatum. Despite the moderate effect of betanin on MPTP-induced dopaminergic damage, our findings confirm the beneficial effect of betanin on the behaviour of mice and reduction in motor deficits induced by the neurotoxin administration—both in the Rota Rod and Footprint tests. The MPTP injected group had an abnormal shortened step length pattern (*p* < 0.05, NK) and a shorter fall time (*p* < 0.05, NIR).

It is likely that the improvement in motor function associated with betanin administration has a mechanism not directly related to the effect on the dopaminergic system. Our observations are consistent with other studies. ElSayed et al. [24] investigated the neuroprotective effect of betanin in rotenone-induced parkinsonian-like motor disorders in mice. Rotenone is a pesticide with a highly selective inhibitory effect on complex I of the mitochondrial respiratory chain. Treatment with betanin at doses of 50 and 100 mg/kg b.w./48 h for 20 days caused a dose-dependent improvement in the crossed squares test, as well as the activity index in open field test. After betanin treatment, a dose-dependent improvements in the fall time in the Rota Rod test and improvement in the fall time in pole test were observed. Improvement in motor function was correlated with significant dose-dependent reductions in striatal inflammatory cytokines such as TNF-α, IL-1β and IL-6 in the experimental group receiving betanin compared with the rotenone group. Furthermore, betanin improved striatal dopamine and GSH levels, but simultaneously caused a decrease in malondialdehyde and TLR4/MyD88/NF-κB protein levels.

Thong-asa et al. [25] confirmed that betanin reverses motor dysfunction and neurodegeneration in a rotenone-induced Parkinson’s disease model. Animals receiving betanin showed improvement in the hanging wire fall time in the Rota Rod test. In mice, betanin at a dose of 100 and 200 mg/kg b.w./48 h prevented the increase in malon-dialdehyde levels and the degeneration of neurons in the substantia nigra, striatum and motor cortex.

In a previous study, Thong-Asa et al. [26] confirmed the neuroprotective effects of betanin in a mouse model of trimethyltin-induced neurodegeneration. It was demonstrated that betanin administered at a dose of 100 mg/kg body weight exhibited anxiolytic properties, improved spatial memory and learning processes, and prevented degeneration of the Cornu Ammonis 1 (CA1) region of the hippocampus. Although betanin is a polar and hydrophilic compound, which generally limits its passive penetration across the blood–brain barrier (BBB), the observed neuroprotective effects in animal models suggest that it may either cross the BBB via active transport mechanisms or exert indirect effects on the central nervous system. The aforementioned study showed that oral administration of betanin significantly attenuated trimethyltin-induced neurodegeneration by enhancing spatial memory, reducing hippocampal damage, and increasing the activity of antioxidant enzymes such as catalase and superoxide dismutase. Moreover, betanin reversed the trimethyltin-induced depletion of glutathione levels, further supporting its antioxidant potential. Despite limited direct evidence regarding betanin’s permeability across the BBB, the accumulated data emphasize its ability to affect brain tissue and highlight the necessity for further research on its pharmacokinetics and therapeutic applications in neurodegenerative diseases such as Parkinson’s disease.

In our study, the main changes in neurotransmitters levels in CNS structures concern dopaminergic and serotonergic systems. Dopamine and serotonin play complementary roles in the processes of reinforcement and control of behaviours related to choice, motivation and planning [27,28]. Serotonin is a neurotransmitter closely related to the maturation and ageing of neurons, as well as control of motor neuron excitability, functional recovery of spinal cord motor neurons, and global regulation of motor behaviour [29,30,31].

The presented studies have shown that betanin at a dose of 50 mg increases serotonin levels in the striatum, prefrontal cortex and hippocampus. Ansah et al. [32] have proven that the neurotoxic effect of MPTP on the dopaminergic and noradrenergic systems of the striatum is age-dependent. In 3-month-old male C57BL/6J mice, MPTP poisoning causes a decrease in dopamine levels in the striatum without changes in 5-HT and NA three weeks after MPTP treatment. During the study, a partial regeneration of DA content in the striatum was observed 18 months later, which was accompanied by an increased level of serotonin in this brain structure. In turn NA in the prefrontal cortex was reduced, but a complete regeneration was observed 18 months later. At the same time, the authors found that striatal dopamine innervation in aged mice was significantly more susceptible to MPTP intoxication, showing more than a 60% decrease in striatal dopamine content compared to young adult mice (a statistically insignificant decrease of about 25%).

In primates, an increase in the number of serotonin axonal processes in the striatum was observed after MPTP administration. Our results are consistent with the observations of other researchers who have shown in animal models that damage to the substantia nigra causes a rapid release of serotonin [33]. It is known that serotonin is responsible for maintaining the balance between dopamine levels in the striatum and the cerebral cortex. Serotonergic afferent fibres in the striatum are known by high plasticity, therefore destruction of the substantia nigra causes a rapid hyperinnervation of the striatum by serotonergic neurons and compensatory processes are initiated, including increased serotonin release in this brain structure [34].

The observed increase in DOPAC/DA turnover in the striatum after administration of a higher dose of betanin, may be related to dopamine release. This effect may be a compensatory mechanism related to the destruction of dopaminergic neurons and reduced dopamine release. Increased serotonin levels in the striatum facilitate the release of dopamine from dopaminergic terminals in the substantia nigra [7,33,35].

At the same time, our study showed that in other CNS structures studied (prefrontal cortex, spinal cord and hypothalamus) after administration of a higher dose of betanin, serotonin content decreases, while the concentration of metabolites of this neurotransmitter and its turnover increase. Betanin at a dose of 100 mg causes an increase in the level of 5-HIAA—the main product of serotonin degradation in the prefrontal cortex, as well as in the spinal cord and cerebellum, which reflects serotonin turnover and metabolism. This indicates that in structures that are not as sensitive to the neurotoxin MPTP, serotonin metabolism is increased, which may be due to activation of betanin-metabolizing enzymes such as monoamine oxidase.

Several studies have confirmed that betanin can alter enzymatic activity. A study conducted by Salimi et al. [36,37] showed that betanin induces apoptosis in human glioma U87MG cells. Intraperitoneal injection of betanin (25 mg/kg b.w.) to rats reveals neuroprotective effects in neurotoxicity induced by cytarabine—an anticancer agent used as a first-line treatment for leukemias and lymphomas. The decrease in the activities of enzymes such as acetylcholinesterase and butyrylcholinesterase in rat brain tissues was attenuated by betanin via modulation of oxidative activity and protective effect mitochondria. At the same time, Salimi et al. [38] showed that betanin at a dose of 25 and 50 mg injected intraperitoneally for 9 days reversed scopolamine-induced memory impairment and attenuated tissue damage and mitochondrial dysfunction. In animals, betanin administration (at a higher dose) improved scopolamine-induced short term memory impairment in a novel object recognition test. At the same time, betanin at a dose of 50 mg improved memory retention during training and passive avoidance test. Histopathological examination of the hippocampus showed that at this dose betanin reduced scopolamine-induced abnormalities such as capillary congestion or neuronal loss in the CA1 area, by reducing the formation of reactive oxygen species in mitochondria.

Thawkar et al. [39] also assessed the neuroprotective properties of betanin in a scopolamine-induced cognitive dysfunction model. Betanin (50, 100, and 200 mg/L) and donepezil (10 mg/L), a drug commonly used to treat Alzheimer’s disease by inhibiting acetylcholinesterase (AChE) and thereby increasing acetylcholine levels in the brain, were administered to zebrafish in a treatment tank once a day for 8 days. Scopolamine was given an hour before the behavioural assessments: the Y-maze and novel tank diving test. The results of these studies indicated that betanin at doses of 50 and 100 mg increased AChE activity, reduced anxiety, and improved memory as well as antioxidant capacity in the brains of animals with scopolamine-induced cognitive dysfunction.

Betanin may alleviate MPTP-induced behavioural disorders in several ways not only through its anti-inflammatory and antioxidant properties, but also through modulation of the endocrine system [40]. However, the results of the current study showed that at proposed doses, betanin is able to reverse the adverse changes in the dopaminergic system caused by MPTP only to a small, limited extent, and additionally reduces serotonin levels in the hypothalamus, a structure that produces several releasing and inhibiting hormones.

The limited ability of betanin to reverse the adverse CNS disorders after MPTP injection may be due to the poor oral bioavailability of this compound and its degradation by intestinal bacteria to the betanidin aglycone after oral administration [41].

Other investigators have shown that chronic MPTP administration causes neuronal loss extending beyond the nigrostriatal dopaminergic system to include brainstem serotonergic and noradrenergic neurons [42,43].

The presented data allow for expanding knowledge on the previously unknown, central action of betanin and its influence on the functions of the central nervous system, including cognitive processes, anxiety and psychomotor functions, as well as for investigating the molecular mechanisms leading to the observed changes in animal behaviour. The obtained data show that betanin does not prevent the loss of dopamine induced by MPTP but significantly changes the level of serotonin in many structures of the central nervous system. It is suggested that the observed beneficial effect of betanin on the mobility and cognitive functions of animals is not directly correlated with the modification of the level of neurotransmitters in the structures of the central nervous system but may be the result of other central and peripheral effects. Understanding the biochemical changes that occur in the brain of animals after long-lasting administration of betanin may also help to determine whether this compound may be important in the prevention or treatment of specific neurodegenerative diseases.

This study shows that betanin can improve motor function impaired by MPTP and reduce neurological damage in an animal model that mimics Parkinson’s disease. Betanin reduces motor deficits and anxiety in mice but does not fully compensate for DA deficits caused by MPTP poisoning. Further, more advanced research of betanin may support its use in preventing neurodegenerative disorders.

## 4. Materials and Methods

### 4.1. Animals and Treatment

Animal experiment was conducted on male C57BL/10/Clzd mice, aged 18 months. Half of animals received betanin (Sigma-Aldrich) in drinking water for a total of 55 days at a dose of 50 mg or 100 mg/kg b.w./day. At the same time, the rest of the animals received drinking water. On the 26th day of betanin administration, some animals were intraperitoneally injected with 1-methyl-4-phenyl-1,2,3,6-tetrahydropyridine (MPTP; Sigma-Aldrich) to establish the PD model.

The mice finally formed four equal groups:

Group 1 (Con)—control animals receiving drinking water and saline intraperitoneally for 5 days (n = 8)

Group 2 (MPTP)—mice receiving drinking water and MPTP 30 mg/kg b.w./day intraperitoneally for 5 days (n = 8)

Group 3 (Bet50 + MPTP)—animals receiving betanin 50 mg/kg b.w./day orally dissolved in drinking water for 55 days and MPTP 30 mg/kg/day intraperitoneally for 5 days (n = 8)

Group 4 (Bet100 + MPTP)—animals receiving betanin 100 mg/kg/day orally in drinking water for 55 days and MPTP 30 mg/kg/day intraperitoneally for 5 days (n = 8)

**Figure 9:**
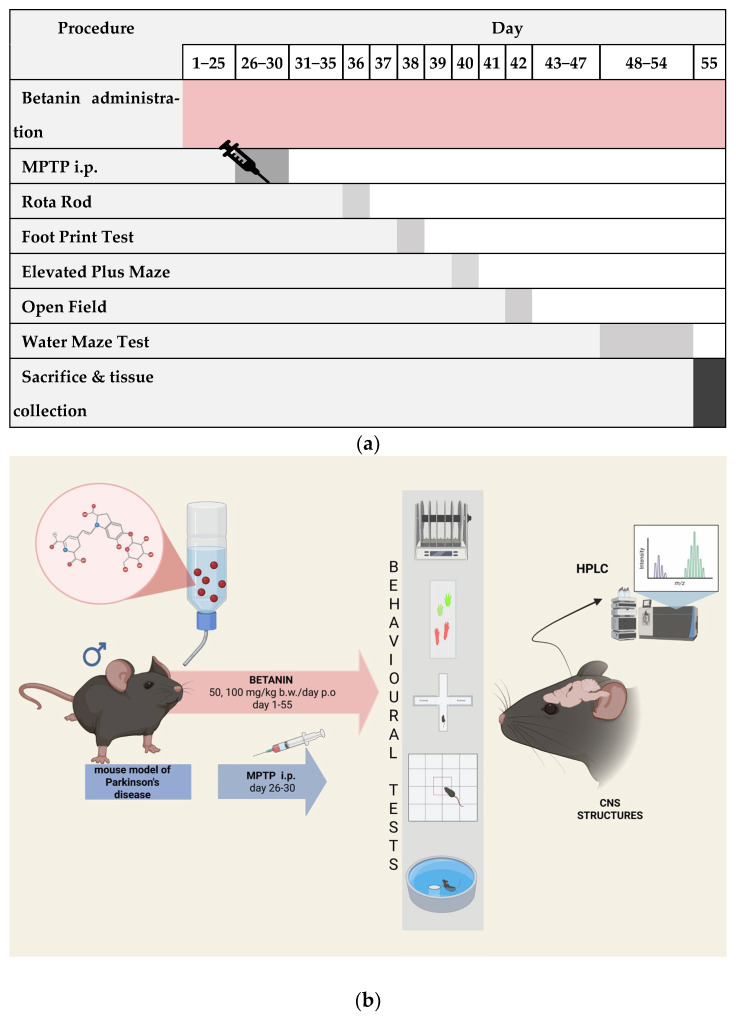
Graphical representation of the experimental procedures (**a**) and the timeline overview of the study (**b**), showing betanin administration (days 1−55), MPTP injections (days 26−30), behavioural tests at specific time points, and sacrifice on day 55 (created with https://www.biorender.com, accessed on 28.09.2025).

Typical symptoms of Parkinson’s disease were induced by administering MPTP for 5 days at a dose 30 mg/kg b.w./day. At the same time, the control group received physiological saline administered intraperitoneally. Betanin administration was continued for the following days, as well as during behavioural tests. Figure 9 shows the graphical representation of the experimental procedures (a) and the timeline overview of the study (b).

All groups of mice were placed individually in plastic breeding cages (4–5 males per cage) in an air-conditioned room with mechanical ventilation (15–20 air changes per hour), a regulated 12 h dark-light cycle, temperature of 20–24 °C and humidity of 55% (±10%). Environmental enrichment in the form of red plastic transparent houses, cotton rolls, and wooden fibres for building a nest were used in all cages.

Animals were provided with standard food (Labofeed, Kcynia, Poland) and had ad libitum access to either drinking water or betanin solution. Daily monitoring included observation of animal activity, visual assessment of general health, and control of feed and fluid consumption. The amount of betanin solution was adjusted as needed based on daily measurements to maintain an approximate intended dose. Body weight was periodically recorded, and daily water consumption per cage was measured to modulate betanin concentration accordingly.

Although individual intake could not be precisely determined due to shared water sources, adult mice typically consume 5–7 mL of water per day, and it was assumed that individual variations balanced out over the prolonged treatment period. This administration method was chosen to minimize stress and better replicate physiological compound intake compared to invasive dosing routes, which is especially relevant considering the potential effects of stress on behavioural outcomes. However, the inability to control exact individual dosing represents a limitation that should be taken into account when interpreting the results.

Animals then participated in behavioural testing. Body weight was recorded at several time points during the experiment and immediately prior to sacrifice by cervical dislocation. Finally, tissues were collected for further analysis.

### 4.2. Behavioural Tests

Animals were trained in a battery of motor and cognitive tests. A graphical abstract of the experiment is shown in Figure 9a,b. To obtain a broad assessment of the animal’s behaviour in terms of cognitive processes, anxiety, as well as exploratory and motor activity, the following behavioural tests were performed:

- Rota Rod—a test assessing motor coordination, balance, strength and endurance;
- Footprint test—a test assessing motor coordination and synchronization (used in the case of movement disorders and gait analysis);
- Elevated Plus Maze—for examining anxiety-related behaviour;
- Open Field—motility and anxiety assessment;
- Water Maze—for studying spatial memory, learning processes and memory consolidation.

Each behavioural test was video recorded and scored with the Noldus EthoVision XT10 system (detection settings: centre-point detection, dynamic subtraction, sample rate: 8.33/s).

#### 4.2.1. Rota Rod Test

The Rota Rod (Accelerating Model, Ugo Basile, Biological Research Apparatus, Varese, Italy) is used to assess motor coordination, balance, and motor skills in animals. Mice were assessed in a device made of a rotating cylinder that forces motor activity. During the training period, each mouse was placed on the Rota Rod for a maximum of 60 s, and the mean latency to fall was recorded and used for subsequent analysis. Mice were subjected to 3 trials per day, with a 10 min break between trials. Each trial started at a constant speed of 5 rpm, which gradually increased to 40 rpm to progressively challenge motor coordination and balance.

#### 4.2.2. Footprint Test

Motor coordination and balance in mice were analyzed using Footprint test. To assess movement patterns, animals were trained to walk along a 60 cm-long, 12 cm wide white paper covered runway (with 13 cm walls) into a closed black box. All mice underwent three training runs during which the animal’s footprints were recorded. To obtain footprints, the hind and forepaws of the animals were covered with red and green nontoxic paints, respectively. For each run, a fresh sheet of paper was placed on the runway floor.

To characterize the gait pattern of each mouse, footprints were analyzed based on three spatial parameters measured in millimetres: stride length (SL), step length (StL), and step width (SW). Stride length (SL) refers to the distance between two consecutive placements of the same limb (e.g., left hind paw), representing the length of a complete step cycle and indicating the forward progression of a limb. In contrast, step length (StL) denotes the distance between the placement of two subsequent limbs on the same side of the body (e.g., from the left forepaw to the left hind paw) and reflects interlimb coordination during locomotion. Step width (SW), defined as the lateral distance between the left and right hind paw placements, serves as an indicator of gait stability and balance. Together, these parameters provide complementary and quantitative information about locomotor patterns, enabling the assessment of motor coordination, balance, and potential gait disturbances.

For each step parameter, three values were measured from each run, excluding footprints made at the beginning and end of the run. Mean value of each set of three values was used in subsequent analysis.

#### 4.2.3. Elevated Plus Maze (EPM) Test

The Elevated Plus Maze (EPM) is a research tool in neurobiological studies of anxiety. To assess anxiety-like behaviour and aversion to open and elevated spaces, mice were tested in an apparatus placed at a height of 50 cm, consisting of four white arms (31 cm × 6.5 cm × 17 cm, L × W × H) connected by a central square. The two arms were surrounded by white sidewalls. The apparatus was located in an acoustically insulated experimental room illuminated by diffuse light (100 lux light placed 1.50 m above the testing apparatus). Animal behaviour: time spent in open and closed arms, number of entries into closed and open arms, total distance, mean speed, and latency to first entry into open arms, were studied during a 5 min session. To eliminate traces of odours, the field was cleaned with 10% ethanol solution before each animal test.

#### 4.2.4. Open Field (OF) Test

The Open Field (OF) test quantifies the spontaneous exploration of animals as well as various locomotor parameters such as total distance travelled or time spent moving. The standard OF apparatus was placed in an acoustically isolated experimental room illuminated with diffused light (100 lux light placed 1.50 m above the test apparatus). The open field was divided into central and peripheral zones. During the test, mice were placed individually in the centre of the freshly cleaned cubic arena (0.57 m × 0.57 m with 0.50 m side walls) made of grey Plexiglass for 5 min with a video camera placed above the OF arena, and the trial was recorded using EthoVision XT10 software ver.10.1.856 (Noldus).The animal’s behaviour was assessed by observers who were blinded to the experimental design. After each trial, the maze was cleaned with 10% ethanol solution.

After analyzing the data, the latency time, time spent in the middle zone, time spent moving and motionless, total distance, speed, climbing, defecation were assessed.

#### 4.2.5. Water Maze (WM) Test

To reduce the stress and the natural aversion of rodents to the aquatic environment, mice were accustomed to the experimental conditions 24 h before testing by swimming for 60 s in a circular pool located in a different room than the one in which the behavioural test was conducted. The water maze test was conducted in a pool with a diameter of 1.2 m, height of 0.50 m, conventionally divided into four quadrants (Southeast—SE, Southwest—SW, Northeast—NE, Northwest—NW) filled with water at a temperature of 23 ± 0.5 ˚C to a height of 0.3 m. Test was conducted in a separate room surrounded by several objects as spatial coordinates and isolated from external sound stimuli.

During the acquisition phase of the Morris Water Maze test, animals underwent repeated trials in which they learned to locate a hidden platform using spatial cues, allowing assessment of their spatial learning ability. The animals swam 4 times on 4 consecutive days (the duration of one swim was max. 60 s) and learned to find a transparent platform (Ø 10 cm) placed 1 cm below the water surface in the centre of the SE quadrant. In each trial, the mouse was placed into the pool facing the wall at equidistant cardinal points in a pseudorandom schedule. As the mouse found the platform, it was allowed to remain there for 15 s to recognize distal cues. If the animal did not find the underwater platform within 60 s, the experimenter manually guided the animal to the platform for 15 s. After the swimming, each animal was removed from the pool, carefully dried with a towel and returned to its cage.

On the fifth day, the platform was removed from the pool, and the mice swam for 60 s in search of the missing platform (memory test). The water maze protocol described above was used to assess the animals’ spatial memory, both learning and memory consolidation, and to assess sensorimotor skills. Sensorimotor skills and motivation were analyzed in a task with a visible platform raised above the water surface (cued task). In this task, the daily training session consisted of 4 trials in which the platform was 1 cm above the water level and moved sequentially within four quadrants. The test was terminated when the animal reached the platform or after 60 s. Latencies to find the platform, number of visits to the target area and time spent in the target quadrant, as well as swimming routes and speed were recorded using a video camera and EthoVision XT10 software (Noldus).

### 4.3. High-Performance Liquid Chromatography Analysis

After completing the behavioural procedures, animals were sacrificed by cervical dislocation. CNS structures were immediately collected, weighed, snap-frozen, and stored at −80 °C until future analysis. High-performance liquid chromatography (HPLC) was used to estimate monoamine and metabolite concentrations in selected mice CNS tissues: hippocampus, prefrontal cortex, striatum, hypothalamus, cerebellum, medulla oblongata and spinal cord.

#### 4.3.1. Sample Preparation

Before analysis, brain samples were homogenized using an ultrasonic cell disrupter (VirSonic 60; VirTis, Gardiner, NY, USA) in a mixture (1000 μL) containing ice-cold 0.1 N perchloric acid (HClO4) and 0.05 mM ascorbic acid, and then centrifuged to precipitate proteins (Heraeus Labofuge 400 R, Heraeus Instruments, Germany; centrifugation conditions: speed of 13,000× *g*, 15 min, 4 °C). After filtration using 0.2 μm syringe membrane filters (Puradisc; Whatman, Maidstone, UK), the supernatant was collected and used for biochemical analyses.

#### 4.3.2. Monoamines Concentration Assay

To determine the concentrations of serotonin (5-HT) and its metabolite 5-hydroxyindoleacetic acid (5-HIAA), dopamine (DA) and its metabolites 3,4-dihydroxyphenylacetic acid (DOPAC) and homovanillic acid (HVA), and noradrenaline (NA) and its metabolite 3-methoxy-4-hydroxyphenylglycol (MHPG) a 20 μL aliquot of the supernatant was injected into the an HPLC apparatus instrument with an electrochemical detection (HPLC-ED) system.

The HPLC system consisted of a delivery pump (Mini-Star K-500; Knauer, Berlin, Germany), an autosampler automatic sample injector (LaChrom L-7250; Merck-Hitachi, Darmstadt/Tokyo, Germany/Japan), and an electrochemical detector (L-3500A; Merck-Recipe, Darmstadt/Munich, Germany) set at a potential of +0.8 V vs. an Ag/AgCl reference electrode. The mobile phase consisted of 32 mM sodium phosphate buffer (Sigma-Aldrich, St. Louis, MO, USA), 34 mM citric acid buffer (Sigma-Aldrich, USA), 1 mM octanesulfonic acid buffer (Sigma-Aldrich, USA), 54 μM ethylenediaminetetraacetic acid (EDTA) buffer (Sigma-Aldrich, USA) in ultrapure water (18 MΩ cm) containing 12% methanol solution (Merck, Germany). Monoamines were separated using EC 250/4 Nucleosil 100-5 C18AB (250 mm length × 4 mm internal diameter, 5 µm particle size, 100 Å), HPLC analytical column (Macherey-Nagel, Germany) and the mobile phase flow rate was maintained at 0.8 mL/min. Chromatograms were recorded and integrated using Clarity computer data acquisition software (version 5.0; DataApex, Prague, Czech Republic). Samples were quantified by comparison with standard solutions (external calibration). All monoamine standards used were purchased from Sigma-Aldrich, USA. The final amount of monoamines/metabolites in the tissue sample was expressed as pg/mg fresh tissue.

### 4.4. Statistical Analysis

Statistical analyses were performed using TIBCO Statistica 13.3. software. Behaviour data from the Open Field, Elevated Plus Maze, Rota Rod, Water Maze, Footprint tests and biochemical analysis of CNS structures were analyzed using analysis of variance (ANOVA) with Newman-Keuls (NK) post hoc test and NIR for multiple comparisons. Results were considered statistically significant at *p* < 0.05. Scatter plots and box plots were used to visualize data.

## Data Availability

The data presented in this study are available on request from the corresponding author.

## Supplementary Materials

The following supporting information can be downloaded at: https://www.mdpi.com/article/10.3390/ijms26199726/s1.

## Abbreviations

The following abbreviations are used in this manuscript:

5-HIAA	5-hydroxyindoleacetic acid
5-HT	serotonin
3-MT	3-metoxytyramine
ANOVA	analysis of variance
CNS	central nervous system
COMT	catechol-O-methyl transferase
CON	control group
DA	dopamine
DOPAC	3,4-dihydroxy-phenylacetic acid
EDTA	ethylenediaminetetraacetic acid
EPM	Elevated Plus Maze
GABA	gamma-aminobutyric acid
HPLC	High Performance Liquid Chromatography
HVA	homovanillic acid
JNK	c-Jun N-terminal Kinase
MPTP	1-methyl-4-phenyl-1,2,3,6-tetrahydropyridine
MHPG	3-methoxy-4-hydroxyphenylglycol
MAO-B	monoamine oxidase B
NA	noradrenaline
NK	Newman-Keuls test
Nrf2	nuclear factor erythroid 2–related factor 2
OF	The Open Field
PD	Parkinson’s disease
PI3K	phosphoinositide 3-kinase
SAPK	stress-Activated Protein Kinase
SER	serine
SEM	standard error of the mean
WM	Water Maze
/c-Jun	N-terminal Kinase (/)
