# Supplementary material for: Neuroprotective Effects of Betanin in a Mouse Model of Parkinson’s Disease: Behavioural and Neurotransmitter Pathway Insights
Source: Int J Mol Sci. 2025 Oct 6;26(19):9726. doi: 10.3390/ijms26199726 (PMC12524363; doi:10.3390/ijms26199726)
Supplement: Supplementary file 1 [file ijms-26-09726-s001.zip › Supplementary Materials Table S1.pdf]

Table S1

Monoamine and metabolite levels (pg/mg ± SEM)	Brain region			
	Group	Prefrontal cortex	Hippocampus	Striatum
NA	Con	**347.14 ±27.03**	295.10±10.15	258,66±28.10
	MPTP	307.34 ±16.50	297.89±35.27	195.17±31.99
	Bet50 + MPTP	**270.99±18.10***	294.77±14.23	264.97±35.52
	Bet100 + MPTP	**251.73±11.55****	285.29±12.05	217.17±16.16
MHPG	Con	n.d.	n.d.	n.d.
	MPTP			
	Bet50 + MPTP			
	Bet100 + MPTP			
DA	Con	169.27±68.27	**661.71±176.00**	**5979.04±797.92**
	MPTP	92.91±15.59	**269.23±120.27***	**2176.06±304.39*****
	Bet50 + MPTP	466.00±209.85	**120.57±19.71****	**1811.96±283.68*****
	Bet100 + MPTP	147.17±3 2.52	**233.99±47.92***	**1772.71±262.30*****
DOPAC	Con	86.27±15.17	64.15±14.52	**446.53±44.24**
	MPTP	60.51±12.12	35.65±17.38	**210.41±31.10*****
	Bet50 + MPTP	120.80±55.55	15.46±2.48	**163.32±26.71*****
	Bet100+MPTP	78.95±24.55	33.73±7.43	**240.71±63.54*****
HVA	Con	121.82 ± 0.58	113.18±19.56	**701.65±84.40**
	MPTP	**79.59±12.06**	89.15±29.63	**456.99±57.61***
	Bet50 + MPTP	**150.86±33.51**□	54.97±7.56	**445.58±42.89***
	Bet100+MPTP	**150.33±9.00**•	93.32±11.13	**440.97±64.10***
3-MT	Con	n.d.	**71.97±14.47**	**214.44±25.73**
	MPTP		48.97±18.49	**182.32±31.86**
	Bet50 + MPTP		**19.22±4.67***	264.58±55.80
	Bet100 + MPTP		**17.04±12.71***	**377.72±52.50***•
5-HT	Con	**452.28±24.46**	**574.14±40.58**	**481.00±29.16**
	MPTP	436.75±18.54	**727.30±58.05**	**450.64±27.45**
	Bet50 + MPTP	**482.89±26.80**$^{\#}$	**862.66±43.57*****■	**593.11±43.87**■$^{\#}$
	Bet100 + MPTP	**385.52±17.59**▲	**635.38±42.28** ▲▲	**558.80±31.17**○
5-HIAA	Con	**226.21±12.00**	307.21±41.65	286.64±30.49
	MPTP	**245.84±10.95**	400.81±32.09	268.03±15.69
	Bet50 + MPTP	**263.42±18.47**	424.31±33.70	291.71±17.70
	Bet100 + MPTP	**307.77±15.52*****▲•	392.07±32.06	303.37±25.33

Table S1

Table S1. Effect of intraperitoneal administration of MPTP and betanin in drinking water on the levels of monoamines and metabolites (mean ± SEM) in brain regions of male mice. Bold font indicates significant differences

* *vs* Con, p<0.05 (NK)
** *vs* Con, p<0.01 (NK)
*** *vs* Con, p<0.005 (NK)
$^{\#}$ *vs* Con, p<0.05 (NIR)
▲ Bet50 + MPTP *vs* Bet100 + MPTP, p<0.05 (NK)
▲▲ Bet50 + MPTP *vs* Bet100 + MPTP, p<0.01 (NK)
▲▲▲ Bet50 + MPTP *vs* Bet100 + MPTP, p<0.005 (NK)
• MPTP *vs* Bet100 + MPTP, p<0.05 (NK)
••• MPTP *vs* Bet100 + MPTP, p<0.005 (NK)
◦ MPTP *vs* Bet100 + MPTP, p<0.05 (NIR)
▪ MPTP *vs* Bet50 + MPTP, p<0.05 (NK)
▫ MPTP *vs* Bet50 + MPTP, p<0.05 (NIR)
n.d.- not detected
