# Supplementary material for: Neuroprotective Effects of Betanin in a Mouse Model of Parkinson’s Disease: Behavioural and Neurotransmitter Pathway Insights
Source: Int J Mol Sci. 2025 Oct 6;26(19):9726. doi: 10.3390/ijms26199726 (PMC12524363; doi:10.3390/ijms26199726)
Supplement: Supplementary file 1 [file ijms-26-09726-s001.zip › Supplementary Materials Table S2.pdf]

Table S2

Monoamine and metabolite levels (pg/mg ± SEM)	Brain region				
	**Group**	**Cerebellum**	**Spinal cord**	**Medulla oblongata**	**Hypothalamus**
**NA**	**Con**	316.35±23.81	197.89±26.88	448.97±36.00	860.66±63.67
	**MPTP**	323.20±14.71	233.97±20.19	448.89±21.87	750.56±58.15
	**Bet50 + MPTP**	310.40±14.86	232.98±14.40	449.38±22.44	888.89±62.18
	**Bet100 + MPTP**	349.87±28.14	225.14±22.89	481.92±30.94	888.46±83.29
**MHPG**	**Con**	n.d.	n.d.	4.38±1.04	n.d.
	**MPTP**			2.44±0.59	
	**Bet50 + MPTP**			3.40±1.05	
	**Bet100 + MPTP**			3.68±1.20	
**DA**	**Con**	18.35±3.16	23.17±2.15	30.03±3.03	**829.55±324.69**
	**MPTP**	24.14±2.90	31.37±5.13	23.35±2.39	**230.09±28.73***
	**Bet50 + MPTP**	19.12±2.21	21.71±1.07	23.67±2.20	**265.00±38.50***
	**Bet100 + MPTP**	26.30±2.70	21.21±2.68	25.50±4.13	**209.37±19.56**$^{\#}$
**DOPAC**	**Con**	n.d.	n.d.	4.39±1.49	**79.13±31.00**
	**MPTP**			1.96±0.61	**18.12±10.26***
	**Bet50 + MPTP**			2.08±0.70	**11.19±2.85***
	**Bet100 + MPTP**			2.55±1.20	**15.17±4.10***
**HVA**	**Con**	n.d.	n.d.	n.d.	**271,95±28.02**
	**MPTP**				**169,32±17.40****
	**Bet50 + MPTP**				**193,47±18.84***
	**Bet100 + MPTP**				**201,97±10.69***
**3-MT**	**Con**	n.d.	n.d.	n.d.	64.61±28.51
	**MPTP**				15.65±5.33
	**Bet50 + MPTP**				9.72±4.24
	**Bet100 + MPTP**				23.79±4.66
**5-HT**	**Con**	291.41±51.67	555.40±17.46	683.63±24.14	**1023.95±16.93**
	**MPTP**	288.21±17.54	**606.17±46.98**	591.08±31.02	922.31±48.03
	**Bet50 + MPTP**	287.45±45.92	**613.39±55.03**	617.52±66.91	**977.60±41.58**
	**Bet100 + MPTP**	376.20±49.02	**441.33±37.08**$^{\blacktriangle \bullet}$	570.25±36.85	**858.88±47.68***$^{\triangle}$
**5-HIAA**	**Con**	166.52±26.35	**180.11±19.95**	361.90±29.65	517.41±36.53
	**MPTP**	166.32±13.05	208.12±12.12	375.72±21.66	562.75±29.76
	**Bet50 + MPTP**	150.23±19.28	231.45±15.76	377.34±30.37	546.74±19.60
	**Bet100 + MPTP**	228.39±23.19	**255.36±22.59***	418.76±47.48	579.74±25.19

Table S2

Table S2. Effects of intraperitoneal injection of MPTP and betanin administration in drinking water on the levels of monoamines and metabolites (mean ± SEM) in brain regions of male mice.

Bold font indicates significant differences
* *vs* Con, p<0.05 (NK)
** *vs* Con, p<0.01 (NK)
*** *vs* Con, p<0.005 (NK)
$^{\#}$ *vs* Con, p<0.05 (NIR)
• MPTP *vs* Bet100 + MPTP, p<0.05 (NK)
••• MPTP *vs* Bet100 + MPTP, p<0.001 (NK)
▲BET50 + MPTP *vs* Bet100 + MPTP, p<0.05 (NK)
▲▲▲ Bet50 + MPTP *vs* Bet100 + MPTP p<0.005 (NK)
$^{\Delta}$ Bet50 + MPTP *vs* Bet100 + MPTP, p<0.05 (NIR)
n.d.- not detected
