# Supplementary material for: Neuroprotective Effects of Betanin in a Mouse Model of Parkinson’s Disease: Behavioural and Neurotransmitter Pathway Insights
Source: Int J Mol Sci. 2025 Oct 6;26(19):9726. doi: 10.3390/ijms26199726 (PMC12524363; doi:10.3390/ijms26199726)
Supplement: Supplementary file 1 [file ijms-26-09726-s001.zip › Supplementary Materials Table S3.pdf]

Table S3

Monoamine turnover (mean ± SEM)	Brain region			
	**Group**	**Prefrontal cortex**	**Hippocampus**	**Striatum**
**DOPAC/DA**	**Con**	**0.87±0.18**	0.12±0.02	**0.086±0.01**
	**MPTP**	0.67±0.06	0.12±0.02	0.10±0.00
	**Bet50 + MPTP**	**0.32±0.04*****	0.14±0.02	**0.09±0.01**
	**Bet100 + MPTP**	**0.48±0.06***	0.16±0.03	**0.13±0.02**$^{*\blacktriangle}$
**HVA/DA**	**Con**	0.37±0.37	0.17±0.06	**0.12±0.0061**
	**MPTP**	0.09±0.09	0.48±0.17	0.22±0.0133
	**Bet50 + MPTP**	0.17±0.17	0.20±0.07	**0.32±0.0801****
	**Bet100 + MPTP**	0.24±0.24	0.35± 0.12	**0.26±0.0122**$^{\#}$
**5-HIAA/5-HT**	**Con**	**0.51±0.04**	0.53±0.05	0.59±0.04
	**MPTP**	**0.57±0.03**	0.55±0.02	0.61±0.05
	**Bet50 + MPTP**	**0.56±0.06**	0.49±0.02	0.50±0.03
	**Bet100 + MPTP**	**0.81±0.06** *** •••▲▲▲	0.63±0.06*	0.55±0.04
**MHPG/NA**	**Con**			
	**MPTP**	n.d.	n.d.	n.d.
	**Bet50 + MPTP**			
	**Bet100+MPTP**			

Table S3. Monoamine turnover (mean ± SEM) in selected structures of the central nervous system in mice after intraperitoneal injection of MPTP and betanin in drinking water.

Bold font indicates significant differences
* *vs* Con, p<0.05 (NK)
** *vs* Con, p<0.01 (NK)
*** *vs* Con, p<0.005 (NK)
$^{\#}$ *vs* Con, p<0.05 (NIR)
▲Bet50 + MPTP *vs* Bet100 + MPTP, p<0.05 (NK)
▲▲▲Bet50 + MPTP *vs* Bet100 + MPTP, p<0.005 (NK)
••• MPTP *vs* Bet100 + MPTP, p<0.005 (NK)
n.d.- not detected
