# Supplementary material for: Neuroprotective Effects of Betanin in a Mouse Model of Parkinson’s Disease: Behavioural and Neurotransmitter Pathway Insights
Source: Int J Mol Sci. 2025 Oct 6;26(19):9726. doi: 10.3390/ijms26199726 (PMC12524363; doi:10.3390/ijms26199726)
Supplement: Supplementary file 1 [file ijms-26-09726-s001.zip › Supplementary Materials Table S4.pdf]

Table S4

Monoamine turnover (mean ± SEM)	Brain region				
	**Group**	**Cerebellum**	**Spinal cord**	**Medulla oblongata**	**Hypothalamus**
**DOPAC/DA**	**Con**			0.14±0.04	0.09±0.01
	**MPTP**	n.d.	n.d.	0.08±0.02	0.06±0.03
	**Bet50 + MPTP**			0.78±0.02	0.05±0.01
	**Bet100 + MPTP**			0.09±0.03	0.08±0.02
**HVA/DA**	**Con**				0.63±0.15
	**MPTP**	n.d.	n.d.	n.d.	0.78±0.07
	**Bet50 + MPTP**				0.82±0.11
	**Bet100 + MPTP**				1.04±0.12
**5-HIAA/5-HT**	**Con**	0.59±0.04	0.32±0.03	**0.53±0.03**	**0.51±0.04**
	**MPTP**	0.59±0.05	0.35±0.02	0.64±0.04	**0.62±0.03**
	**Bet50 + MPTP**	0.59±0.09	0.39±0.03	0.66±0.07	**0.57±0.02**
	**Bet100 + MPTP**	0.64±0.06	0.59±0.04 ***•••▲▲▲	**0.73±0.04***	**0.69±0.06***Δ
**MHPG/NA**	**Con**			181.09±47.56	
	**MPTP**	n.d.	n.d.	1195.02±1037.28	n.d.
	**Bet50 + MPTP**			1013.10±593.81	
	**Bet100 + MPTP**			3277.83±2583.89	

Table S4. Monoamine turnover (mean ± SEM) in selected structures of the central nervous system in mice after intraperitoneal injection of MPTP and betanin in drinking water.

Bold font indicates significant differences
* *vs* Con, $p<0.05$ (NK)
*** *vs* Con, $p<0.005$ (NK)
\# *vs* Con, $p<0.05$ (NIR)
▲▲▲Bet50 + MPTP *vs* Bet100 + MPTP, $p<0.005$ (NK)
Δ Bet50 + MPTP *vs* Bet100 + MPTP, $p<0.05$ (NIR)
n.d.- not detected
